# Descriptive Study of Pericarditis Outcomes in Different Etiologies and Risk Factors: A Retrospective Record Review

**DOI:** 10.7759/cureus.27301

**Published:** 2022-07-26

**Authors:** Waddah Y Ashram, Sedrah K Talab, Ruba M Alotaibi, Reem W Baarma, Zainab A Al Nemer, Malak A Alshareef, Halah H AlGhamdi, Reema K Alsubhi

**Affiliations:** 1 Department of Medicine, King Abdulaziz University Hospital, Jeddah, SAU; 2 Department of Medicine, King Abdulaziz University Faculty of Medicine, Jeddah, SAU

**Keywords:** post-covid-19, pericardial friction rub, chest pain, idiopathic pericarditis, pericardial effusion, cardiac tamponade, pericarditis

## Abstract

Background: Pericarditis is an inflammatory pericardial disorder that can be caused by several infectious and non-infectious illnesses. Coronavirus disease 2019 (COVID-19) was recently added to the long list of pericarditis causes. As a result, this study aims to look at the incidence of various etiologies of pericarditis, including post-COVID-19 vaccine and risk factors, at King Abdulaziz University Hospital in Jeddah, Saudi Arabia.

Methods: Between 2012 and 2022, all male and female patients diagnosed with acute, chronic, or constrictive pericarditis at the King Abdulaziz University Hospital clinic were included in this retrospective study, which took place in June 2022. Data were collected from the hospital's medical records, including the patient's demographic information, pericarditis history, medical history, social background, laboratory tests, Echocardiogram (ECHO) and electrocardiogram (ECG) readings, and medication history. Associations were tested using univariate and bivariate analysis.

Results: Acute pericarditis was diagnosed in 59 (89.1%) patients and the most common symptoms were chest pain and shortness of breath (SOB) followed by fever and cough.Idiopathic pericarditis was the primary etiology 30 (46.9%) with male predominance 25 (55.6%), followed by infections and then cardiac presenting primarily with chest pain 25 (83.3%). In comparison, the most common presentation in females was autoimmune, as seen in eight patients (42.1%). Most patients required aspirin, ibuprofen, and colchicine. Among outcomes, of a total of 64 patients, five died within 30 days. Moreover, four (7.5%) experienced subsequent cardiac tamponades, which was mainly due to malignancy (50%) (p<0.05).

Conclusion: There was a substantial relationship between malignancy and developing morbid complications, with 59 patients out of 64 getting acute pericarditis and the remainder chronic and constrictive pericarditis with idiopathic pericarditis being the leading causes.

## Introduction

Pericarditis is a non-ischemic common inflammatory disease of the pericardium that can have an acute or chronic presentation and cause pericardial effusion and constrictive pericarditis [1]. Furthermore, pericarditis may be caused by various infectious and non-infectious underlying pathologies and, very recently, coronavirus disease 2019 (COVID-19) was added to the long list of pericarditis etiology, cardiovascular events,especially pericardial/myocardial injury/myocarditis,* *arrhythmias,​​​​​​​thromboembolic episodes*, *acute coronary syndromes,* *heart failure, and cardiomyopathywere mentioned as consequences of COVID-19* *[2,3].

Acute pericarditis usually presents with very specific chest pain, accounting for 5% of chest pains presented in ER visits. Still, it can also present with vague, non-specific features, including general malaise, diarrhea, and fever [2,4]. In rare cases, severe inflammation can stimulate symptomatic arrhythmias, myocardial infarction, and heart failure. Moreover, cardiac tamponade and sudden cardiac death can be the final consequences of untreated severe acute pericarditis [2]. Diagnosing pericarditis depends mainly on the clinical presentation of chest pain and pericardial friction rub as well as electrocardiogram (ECG) and echocardiogram (ECHO) [4,5].

A prior study in the United States showed that idiopathic pericarditis is responsible for 80-90% of cases. On the other hand, the leading cause in developing countries is tuberculosis (TB), seen in 70% of cases [6,7], which is often associated with human immunodeficiency virus (HIV) infection, especially in sub-Saharan Africa [6,8]. 

Although there are no significant differences in clinical outcomes based on sexes, there was a significant correlation with age, where patients older than 45 had a higher mortality rate (p< 0.001) [9,10]. Additionally, out of 1574 of those who received the second dose of the COIVD-19 vaccine, 161 them got pericarditis in 28 days [11]. Another study showed that 269 patients out of 4,931,775 developed myopericarditis within 28 days of vaccination [12]. However, the prevalence of new-onset pericarditis in adults is not well investigated.

Therefore, this study aims to investigate the prevalence of the different etiologies of pericarditis, including the post-COVID-19 vaccine and risk factors in the time period of 2012-2022 in King Abdulaziz University Hospital, Jeddah, Saudi Arabia.

## Materials and methods

Study design and participants

This retrospective record review study was conducted in 2022 at King Abdulaziz University Hospital, a tertiary center in Jeddah, Saudi Arabia. It was conducted in the department of internal medicine and approved by the Unit of Biomedical Ethics Research Committee of King Abdulaziz University Hospital (approval number 153-22). The cardiology medical records reviewed were from 2012 to 2022 and accordingly, we adjusted the number of participants from the King Abdulaziz University Hospital database. Because of the nature of the study, informed consent was waived.

Data collection

We descriptively analyzed patient demographics data (name, current age, age at diagnosis, gender, nationality, height, and weight), clinical presentation findings (chest pain, cough, dysphagia, palpitation, fatigue, shortness of breath (SOB), orthopnea, paroxysmal nocturnal dyspnea (PND), edema, and fever), physical examination (tachycardia, tachypnea, and pericardial friction rub), type of pericarditis (acute, constrictive, and chronic pericarditis), recurrence of pericarditis, comorbidities (hypertension (HTN), diabetes mellitus (DM), hyperlipidemia, chronic kidney disease (CKD), ischemic heart disease (IHD), congestive heart failure (CHF), chronic liver disease (CLD), rheumatoid arthritis, systemic lupus erythematosus (SLE), and obesity), smoking status, lab investigations (complete blood count (CBC), inflammatory markers, renal function test, liver function test, autoimmune antibodies, infection status (HIV, TB, COVID-19, hepatitis B and C) and uric acid), imaging findings (ECHO, ECG), underlying etiologies (infectious causes, autoimmune causes, cardiac diseases, idiopathic causes, traumatic causes, uremic causes, and malignancy), management “pharmacological agents” (colchicine, ibuprofen, proton pump inhibitors (PPI), and acetylsalicylic acid), and complications (cardiac tamponade and 30 days mortality).

Inclusion and exclusion

The sample comprises all male and female patients diagnosed with acute, chronic, or constrictive pericarditis at our facility between 2012 and 2022. Patients who were diagnosed with pericardial effusion or cardiac tamponade alone were excluded.

Data analysis

Data were entered using Microsoft Excel 2020 (Microsoft Corporation, Redmond, Washington, United States). IBM SPSS Statistics for Windows, Version 21.0 (Released 2012; IBM Corp., Armonk, New York, United States) was used for statistical analysis. Mean and standard deviation were used for numbers and percentages for categorical and continuous variables. Student’s t-test and chi-square test were used to evaluate the differences between the continuous and categorical variables, respectively. Statistical significance was set at p <0.05.

## Results

Univariate

The goal of this study was to determine the prevalence of various etiologies of pericarditis as well as post-COVID-19 vaccine-associated pericarditis. This retrospective study included 64 patients, 45 (70.3%) of whom were males and 19 (29.7%) were female with a mean age of 38.24±15.65 and 47.26±17.41, respectively. Of these, 48.4% fell within the normal body mass index (BMI) range. Acute pericarditis was diagnosed in 59 (89.1%) patients, while the rest were chronic and constrictive pericarditis. The remaining demographics and comorbid conditions are identified in Tables 1, 2.

**Table 1:** Baseline characteristics of studied patients BMI: body mass index

Variables (n= 64)	n (%)
Gender	
Male	45 (70.3%)
Female	19 (29.7%)
Nationality	
Non-Saudi	29 (45.3%)
Saudi	35 (54.7%)
Age (years), Mean±SD	40.92±16.58
BMI	
Underweight	1 (1.6%)
Normal weight	30 (48.4%)
Overweight	18 (29.0%)
Obese class 1	9 (14.5%)
Obese class 3	4 (6.5%)
Smoking status	
Smoker	14 (23%)
Non-smoker	47 (77%)
Pericarditis	
Acute	59 (92.2%)
Chronic	3 (4.7%)
Constrictive	2 (3.1%)

**Table 2:** Comorbid conditions of studied patients DM: diabetes mellitus; HTN: hypertension; IHD: ischemic heart disease; CKD: chronic kidney disease; CHF: congestive heart failure; CLD: chronic liver disease; RA: rheumatoid arthritis; SLE: systemic lupus erythematosus

Variables (n= 64)	n (%)
DM	16 (25%)
HTN	19 (29.7%)
Dyslipidemia	5 (7.8%)
IHD	10 (15.6%)
CKD	9 (14.1%)
CHF	5 (7.8%)
RA	1 (1.6%)
SLE	6 (9.4%)
Obesity	13 (20.9%)
Malignancy	4 (6.3%)

The study reviewed each patient's clinical picture prior to diagnosis and the majority of the symptoms were chest pain (n=51; 79.7%), SOB (n=30; 46.9%), fever (n=20; 31.3%, and cough (n=15; 23.4%). Accordingly, the most common sign was tachycardia (n=16; 25%). Idiopathic pericarditis was the main etiology (n=30; 46.9%), followed by infectious diseases (n=18; 28.1%), then cardiac (n=13; 20.3%), while post-COVID-19 vaccine was only in two patients (3.1%).

Leukocytosis was observed in 17 (27%) patients, whereas leukopenia only presented in six (9.5%). The remaining had normal WBCs, high c-reactive protein (CRP) in 36 (85.7%) cases, and high erythrocyte sedimentation rate (ESR) in 16 (47.1%). In 35 (64.8%) of 64 patients, ECHO demonstrated pericardial effusion while ECG revealed widespread ST elevation in 24 (68.6%).

Bivariate

Males mostly presented with idiopathic pericarditis (n=25; 55.6%), while the most common presentation in females was autoimmune in eight females (42.1%). Subjects with idiopathic pericarditis presented primarily with chest pain (n=25; 83.3%) followed by SOB (n= 10; 33.3%), similar to the cardiac causes. On the other hand, those with infectious causes or post upper respiratory tract infection mainly presented with chest pain (n=15; 83.3%) and fever(n=10; 55.6%).

In addition, 14 (21.9%) of the total sample were known to be smokers, seven (23.3%) were idiopathic, followed by infectious causes in four (22.2%). Moreover, inflammatory markers, including CRP and ESR, were mainly elevated in idiopathic pericarditis (n=15; 88.23%) and (n=7; 23.3%), respectively, as well as leukocytosis observed in nine (30%), next to low hemoglobinemia (Hb) in 17 (56.7%).

Twelve patients who had infectious causes of pericarditis had low hemoglobin and three of them also had leukopenia, while three patients with autoimmune causes of pericarditis had only leukopenia (Table 3). Furthermore, the relation of ECHO with etiology is shown in Table 4.

**Table 3:** Bivariate statistics of etiological laboratory findings WBC: white blood cell; HB: hemoglobin; ESR: erythrocyte sedimentation rate; CRP: C-reactive protein; COVID-19: coronavirus disease 2019 * Statistically significant

Variables	Low, n (%)	Normal, n (%)	High, n (%)	P value (<0.05)
Infectious (n= 18)				
WBC	3 (16.6%)	11 (61.1%)	4 (22.2%)	0.519
HB	12 (66.6%)	6 (33.3%)	0	1.000
ESR	1 (10%)	4 (40%)	5 (50%)	0.308
CRP	1 (7.7%)	1 (7.7%)	11 (84.6%)	0.519
Cardiac (n= 13)				
WBC	1 (7.7%)	9 (69.2%)	3 (23.1%)	1.000
HB	8 (61.5%)	4 (30.7%)	1 (7.7%)	0.285
ESR	1 (33.3%)	1 (33.3%)	1 (33.3%)	0.088
CRP	1 (14.3%)	0	6 (85.7%)	0.190
Autoimmune (n= 8)				
WBC	3 (37.5%)	4 (50%)	1 (12.5%)	0.042*
HB	8 (100%)	0	0	0.089
ESR	0	4 (57.1%)	3 (42.9%)	1.000
CRP	0	1 (14.3%)	6 (85.7%)	1.000
Uremic (n= 7)				
WBC	0	4 (57.1%)	3 (42.9%)	0.464
HB	6 (85.7%)	1 (14.3%)	0	0.473
ESR	0	1 (33.3%)	2 (66.7%)	0.636
CRP	0	1 (16.7%)	5 (83.3%)	0.629
Malignant (n= 4)				
WBC	0	3 (75%)	1 (25%)	1.000
HB	3 (75%)	1 (25%0	0	1.000
ESR	0	1 (100%)	0	0.597
CRP	0	0	1 (100%)	1.000
Post Covid-19 vaccine (n= 2)				
WBC	0	2 (100%)	0	1.000
HB	0	2 (100%)	0	0.135
ESR	0	1 (100%)	0	1.000
CRP	0	1 (50%)	1 (50%)	0.268
Idiopathic (n= 30)				
WBC	1 (3.4%)	19 (65.5)	9 (31%)	0.315
HB	17 (56.7%)	13 (43.3%)	0	0.114
ESR	0	7 (50%)	7 (50%)	1.000
CRP	0	2 (11.8%)	15 (88.23%)	1.000

**Table 4:** Bivariate analysis of etiological echocardiogram findings COVID-19: coronavirus disease 2019

Variable	Pericardial effusion, n (%)	P Value (<0.05)
Infections (n=18)	11 (61.1%)	0.936
Autoimmune (n=8)	6 (75%)	0.698
Cardiac (n=13)	6 (46.2%)	0.728
Uremic (n=7)	5 (71.4%)	0.408
Malignant (n=4)	4 (100%)	0.285
Post COVID-19 vaccine (n=2)	1 (50%)	1.000
Idiopathic (n=30)	14 (46.6%)	0.545

Most patients required aspirin (n=28; 43.8%) followed by ibuprofen (n=20; 31.3%) and colchicine (n=20; 31.3%). Sixteen of the patients received a dosage of 400MG of ibuprofen, two received 600MG, and two received 800MG, with a mean duration of 6.15±4.19. Doses of colchicine were not documented in approximately 68.8% of the patients, yet it was documented in 29.7% of the cases, and they were given 500MCG twice a day with a mean duration of 41.25±41.7.

As outcomes of a total of 64 patients, five died within 30 days. Two among those with cardiac etiology (15.4%), two among the ones with infectious causes (14.3%), and two among the patients with idiopathic etiologies (7.1%) where most of them were within normal weight (80%) followed by overweight. Moreover, four (7.5%) experienced subsequent cardiac tamponades, two of who had malignancy etiologies (50% of the patients with malignancy) with a p-value of (p<0.05) and two from the patients with cardiac etiologies in two (15.4% of the patients with cardiac issues). Regarding BMI, obesity class three was a dominant factor for cardiac tamponade. Finally, subjects with CKD suffered the most complications as three (50%) of them died within 30 days and one (16.6%) developed tamponade.

## Discussion

Pericardial disease is an inflammation of the pericardium. The diagnosis is based on clinical, laboratory, and imaging findings. The goal of this study was to determine the prevalence of various etiologies of pericarditis as well as the post-COVID-19 vaccine pericarditis at King Abdulaziz University Hospital from 2012 to 2022.

To begin with, the majority of pericarditis patients were males 45 (70.3%), similar to the research by Laufer-Perl et al. [10]. In addition, the predominant etiology was idiopathic (46.9%). This supports the results of the studies by Gouriet et al. and Awan et al. [13,14]. Chest pain was the main presentation in the patients, just as was suggested by the two previous studies [13,14]. The significance of this is to build a high suspicion index for all patients with chest pain and clinical findings of pericarditis regardless of their underlying comorbidity status. Moreover: unlike other studies [14], the percentage of pericardial friction rub was only found in three (4.7%), which could most probably be related to the small sample size in our study.

Elevated inflammation markers have always been recommended to aid with diagnosing and monitoring pericarditis. CRP levels were measured in 42 patients and were high in 36 (56.3%) at presentation. Many other studies also mentioned the early elevation of CRP [15-17]. In our study, high CRP levels were mainly seen with idiopathic pericarditis than with infectious causes, being present in 88.23% of patients with idiopathic pericarditis and 84.66% of patients with infectious pericarditis.

One of the major and most important criteria for pericarditis is pericardial effusion. In our study, uremic pericarditis was associated with the highest percentage of pericardial effusion in five (83.3%). It is thought that pericardial effusion is mainly caused by pericardial inflammation. Nevertheless, volume overload also plays a major role, especially in the presence of other signs such as pleural effusion [18]. In addition to pericardial effusion, widespread ST-segment elevation is also observed in 17 (48.6%) of the patients.

While complications of pericarditis can range from recurrence of pericarditis to the life-threatening cardiac tamponade, recurrence was identified as the most common complication by the literature. In our study, recurrence was only observed in seven (10.9%) patients [19]. Moreover, the development of cardiac tamponade was the most concerning complication if not diagnosed and treated successfully, and we found that the development of cardiac tamponade was significantly associated with malignancy (p<0.05), as the study by Fraser et al. suggests that this relation can be explained anatomically due to the lymphatic drainage [20].

Finally, our study had many limitations including the small sample size due to its unicentric nature, the poor documentation of follow-up and outcomes, and possible recall bias. Therefore, we recommend a multicentric study with a large sample size to be conducted prospectively.

## Conclusions

We investigated the prevalence of various etiologies of pericarditis and identified risk factors. In our study, 89.1% of patients had acute pericarditis, with idiopathic being the most prevalent etiology, followed by infectious and cardiac causes. Chest pain was the most common symptom and tachypnea was the most prevalent sign. In addition, 64.8% of patients had pericardial effusion and 37.5% had widespread ST elevation on ECG. Finally, of a total of 64 patients, five died within 30 days, while the main post-pericarditis consequences were of cardiological origin.
